# *Malva sylvestris* leaf powder as a feed additive affects the performance, carcass traits, meat quality attributes, serum antioxidants, stress physiology, intestinal bacterial counts, and gut morphology of broiler chicken

**DOI:** 10.3389/fphys.2024.1462018

**Published:** 2024-09-25

**Authors:** Mohammad T. Banday, Manzoor A. Wani, Haifa A. Alqhtani, May Bin-Jumah, Hassan A. Rudayni, Ahmed A. Allam, Uthman Balgith Algopishi, Sheikh Adil

**Affiliations:** ^1^ Division of Livestock Production and Management, Faculty of Veterinary Sciences and Animal Husbandry, Srinagar, India; ^2^ Department of Biology, College of Science, Princess Nourah bint Abdulrahman University, Riyadh, Saudi Arabia; ^3^ Department of Biology, College of Science, Imam Mohammad Ibn Saud Islamic University, Riyadh, Saudi Arabia; ^4^ Department of Zoology, Faculty of Science, Beni-suef University, Beni-suef, Egypt; ^5^ Department of Biology, College of Science, King Khalid University, Abha, Saudi Arabia

**Keywords:** Malva, phytogenic, feed additive, broiler chicken, performance

## Abstract

This study investigated the effect of supplementation of *Malva sylvestris* leaf powder (MSLP) on the production performance of broiler chicken. Ven Cobb broiler chicks (240 day-old male chicks) were distributed randomly into four treatments, each replicated four times, with 15 birds per replicate. The diets formulated were T1 (control) given basal diet only, T2 (basal diet +1.0% MSLP), T3 (basal diet +1.5% MSLP), and T4 (basal diet +2.0% MSLP). The highest improvement of 3.83% in the average daily gain (ADG) was recorded in the T3 group fed 1.5% *Malva* powder in the diet compared to the control (*P* = 0.009). The average daily feed intake (ADFI) tended to decrease with an increase in the dose of MSLP in the diet, with the lowest feed intake in the T4 group fed 2% MSLP. During the overall period (7–42 days), the feed/gain (F/G) ratio reduced significantly (*P* = 0.048) in the T3 and T4 groups compared to the control. The dressed and breast meat yield was found to be significantly (*P* < 0.05) higher in the T3 group, with no significant change (*P* > 0.05) in the thigh yield. The changes in the pH and water-holding capacity (WHC) of breast meat were found to be non-significant (*p* > 0.05) between the control and various other treatments. Thiobarbituric acid-reactive substances (TBARS) were significantly (*P* < 0.05) decreased in the T3 and T4 groups. There was no negative effect of including MSLP in the diet on the color coordinates of breast meat among different treatments. Compared to the control, the serum immunoglobulin values increased significantly (*P* < 0.05) in the T3 and T4 groups. Superoxide dismutase (SOD) showed no difference between various treatments; however, malondialdehyde (MDA) decreased significantly (*P* < 0.05) according to dietary treatments. Serum cortisol increased significantly (*P* < 0.05) in the T4 group compared to other treatments. The inclusion of *Malva* powder in the diet at the 2% level significantly (*P* < 0.05) decreased the coliform count compared to the control birds. Supplementation with *Malva* powder resulted in a significant *(P < 0.05)* increase in the villus height-to-crypt depth (VH:CD) ratio of broiler birds in the T3 and T4 groups. In conclusion, MSLP supplementation at 1.5% and 2% resulted in improved production performance of broiler chicken.

## 1 Introduction

Due to the notable increase in global population, there is a greater demand for poultry products, which must be supplied while preserving their quality and safety (Abreu et al., 2023). Feed additives are frequently utilized to improve the health and productivity of poultry and produce high-quality meat and eggs (Pirgozliev et al., 2019). Antibiotic growth promoters (AGPs) have been fed to poultry for 50 years, increasing the birds’ overall performance and productivity (Tajodini et al., 2015). However, it is known that their non-selective use could lead to the development of antibiotic-resistant bacteria in the gut of poultry birds. These bacteria can then spread to other hosts, which could result in the genetic antibiotic resistance from poultry being transferred to human microbiota (Sugiharto, 2016; Gadde et al., 2017). In view of this, antibiotic resistance was identified by the World Health Organization as a global public health risk, and in 2006, the European Union also outlawed the use of any antibiotics as feed additives in animals intended for food (Ducatelle et al., 2018; Tajodini et al., 2015). However, eliminating AGPs from poultry diets has resulted in challenges in feed efficiency and growth performance (Sheiha et al., 2020). Therefore, researchers around the world are currently looking for safe feed additives to replace AGPs in poultry diets (Banday et al., 2023). A few of these substitutes are aquatic weeds (Ara et al., 2018; Zaffer et al., 2021), prebiotics (Yaqoob et al., 2021), prebiotics (Anadon et al., 2019), organic acids (Adil et al., 2010), enzymes (Yousuf et al., 2012), and phytobiotics (Adil et al., 2020; Adil et al., 2023; Adil et al., 2024).

Phytobiotics or phytogenic feed additives (PFAs) include herbs, botanicals, essential oils, and oleoresins (Ogbuewu et al., 2020). PFAs have garnered more interest recently as a natural substitute for AGPs in poultry production and may be added to feed in dried, solid, ground, and extract forms (Gadde et al., 2017). A wide range of herbs and spices, including thyme, oregano, cinnamon, rosemary, mint, garlic, ginger, green tea, black cumin, and coriander, as well as essential oils (derived from thymol, carvacrol, lavender, rosemary, and cloves), have been used in poultry, either alone or in combination, as potential application as AGP alternatives (Gadde et al., 2017; Adil et al., 2023; Adil et al., 2024; Ogbuewu et al., 2020; Khursheed et al., 2017). Phenolics, tannins, glycosides, and alkaloids are the primary active ingredients found in phytobiotics (Zhu, 2020). Numerous studies on broilers have shown that PFAs enhance intestinal functions, such as the digestive process and the functioning of the gut microflora, retention of nitrogen in the intestines, the digestibility of fiber, reduction of inflammation, antioxidant activities, immune modulation, and antimicrobial effects, all of which lead to improved overall performance and health (Adil et al., 2023; Adil et al., 2024; Park and Kim, 2018).

*Malva sylvestris*, also known as common mallow in Europe, Iran, Pakistan, and India, is a member of the Malvaceae family of medicinal plants. It is a biennial–perennial herbaceous plant that is frequently found in North Africa, Europe, and Southwest Asia (Alizadeh and Khodavandi, 2016; Barros et al., 2010). It usually thrives in wet environments next to rivers, ditches, marshes, oceans, and meadows (Razavi et al., 2011). It provides many health advantages against a range of ailments and is a rich source of phytochemical compounds (Sabir and Rocha, 2008; Marouane et al., 2011). According to Tabaraki et al. (2012), antioxidants, polyphenols, vitamins C and E, beta-carotene, and other chemical components are responsible for the biological activity of *Malva sylvestris* leaves. Including *Malva sylvestris* in the diet has been reported to improve the growth and intestinal histomorphology of broiler chicken owing to *Malva*’s anti-inflammatory and antimicrobial properties (Kiani et al., 2015). Keeping in view the beneficial effects of *Malva sylvestris*, a study was conducted with the objectives of evaluating the effects of supplementation of *Malva sylvestris* leaf powder on the performance, carcass traits, meat quality attributes, serum antioxidants, stress physiology, intestinal bacterial counts, and gut morphology of broiler chicken.

## 2 Materials and methods

### 2.1 Material and its analysis

The herb *Malva sylvestris* was gathered from various areas of Kashmir Valley, India. The herb’s identity was confirmed at the Department of Environmental Science SKUAST-Kashmir. The leaves were separated and sun-dried in a shaded area. The dried leaves were ground into a fine powder in the analytical laboratory of the Division of LPM, FVSc & AH, SKUAST-Kashmir, and stored until use. The suggested procedures of the Association of Official Agricultural Chemists (AOAC) (AOAC, 2005) were followed for the proximate analysis of *Malva sylvestris* for dry matter (DM), crude protein (CP), ether extract (EE), crude fiber (CF), and total ash (TA). The tests were performed in triplicate, and means were calculated accordingly.

### 2.2 Birds and experiment design

Male VenCobb broiler chicks, 240 days old, weighing an average of 45 ± 2.3 g, were obtained from a local commercial hatchery and brought to the experimental unit of the Division of LPM, FVSc & AH, SKUAST-Kashmir, India. The chicks were kept together for a week, weighed, and then, at random, assigned to 16 floor pens with sawdust as the litter material. There were four treatments and four replications in the completely randomized design with 15 birds per replicate. Four iso-caloric and iso-nitrogenous diets were formulated, viz., T1 (Control) given basal diet only, T2 (basal diet +1.0% MSL), T3 (basal diet +1.5% MSL), and T4 (basal diet +2.0% MSL). The main components of these diets were maize and soybean. National Research Council (NRC) recommendations were followed in the formulation of the basal diet offered to broiler chickens (Soltani and Nobakht, 2016). Table 1 lists the ingredients and nutrient composition of the basal diet. The birds had *ad libitum* access to fresh water and feed. The temperature of the poultry house was maintained initially at 33°C and dropped by 3°C each week until it reached 24°C. Until the completion of the trial, the birds were kept in a photoperiod consisting of 23 h of light and 1 h of darkness. The experiment was carried out in a very hygienic setting, with careful adherence to the vaccination schedule (Newcastle and Gumboro’s diseases on 5th and 14th day post-hatch, respectively) and biosecurity precautions.

**TABLE 1:** Ingredients and nutrient composition of broiler chicken diets.

Ingredients (g/kg)	Starter (7–21 days)	Finisher (22–42 days)
Maize (9.5%)	57.02	59.27
Soybean meal (48%)	30.30	31.24
Fish meal (55%)	6.40	2.00
Vegetable oil	3.20	4.00
Limestone	0.70	1.00
Di-calcium phosphate	1.50	1.60
Salt	0.30	0.30
DL-Methionine	0.10	0.10
Lysine	0.13	0.14
Trace mineral premix^a^	0.10	0.10
Vitamin premix^b^	0.15	0.15
Choline chloride	0.05	0.05
Toxin binder	0.05	0.05
Total	100	100
Analyzed nutrient		
Crude protein (%)	21.60	19.99
Calculated nutrient content		
Metabolizable energy (Kcal/kg)	3,080	3,154
Calcium	1.09	1.05
Available P	0.47	0.44
Lysine	1.26	1.16
Methionine	0.50	0.44

^a^ Trace mineral premix (mg/kg diet): Mg 300, Mn 55, I 0.4, Fe 56, Zn 30, and Cu 4.

^b^ Vitamin premix (per kg diet): vitamin A 8250 IU, vitamin D3 1200 ICU, vitamin K 1 mg, vitamin E 40 IU, vitamin B1 2 mg, vitamin B2 4 mg, vitamin B1 210 mg, niacin 60 mg, pantothenic acid 10 mg, and choline, 500 mg.

### 2.3 Performance evaluation

The birds in each replicate were weighed at the start and each week during the trial. Each replicate’s average daily gain (ADG), average daily feed intake (ADFI), and feed-to-gain (F/G) ratio were calculated by recording feed intake and the number of surviving birds in that replicate.

### 2.4 Collection of samples

On the 42nd day of the experiment, two birds per replicate were chosen at random, and blood samples were taken from the wing vein for analysis of serum immunoglobulins and antioxidant enzymes. The Division of Livestock Products Technology, FVSc & AH, SKUAST-Kashmir slaughterhouse used the Halal procedure to slaughter each chicken (two birds per replicate). Each bird was weighed immediately prior to severing the jugular vein at the atlanto-occipital joint and allowing it to bleed. The shanks were severed at the hock joint, and the carcass was scalded at 60°C for 30 s. The feathers were removed entirely by handpicking, leaving the skin intact. The abdominal cavity was then opened to expose the visceral organs, and the carcass features were assessed. The contents of the cecum were promptly gathered into sterile polybags and kept at −20°C for microbiological analysis. The jejunum samples, measuring approximately 2 cm in length, were cut using scissors, and after being thoroughly cleaned in physiological saline to remove all the contents, they were preserved in 10% buffered formalin solution for further histomorphological analysis.

### 2.5 Carcass and meat quality traits

The dressing percentage, weight of breasts, thighs, and abdominal fat percentage of the slaughtered birds were recorded. The breast meat samples were used to assess meat quality. The pH levels of meat samples after slaughter were ascertained. In order to do this, 10-g sections of the samples were combined with distilled water and homogenized in a homogenizer for 1 min. A digital pH meter (Tanco, India) was used to measure the pH readings. Additionally, the water-holding capacity (WHC) of the minced meat sample was estimated by mixing it with 0.6 M NaCl, holding it at 4°C for 15 min, and measuring the supernatant fluid after centrifugation at 5,000 rpm for 15 min (Brondum et al., 2000). The color coordinates (lightness-L*, redness-a*, and yellowness-b*) of the cross-sectional sections of meat samples were determined using a colorimeter (YS3060). For every sample, three color measurements were made directly on the muscle’s surface at various points, and the results were averaged. The assessment of meat samples’ lipid peroxidation was conducted by measuring the amount of thiobarbituric acid-reactive substances (TBARS). The TBARS value was estimated by slightly altering the method of Witte et al. (1970). Initially, 25 mL of 20% trichloroacetic acid (TCA) that had been pre-cooled was added to 10 g of material, and it was triturated for 2 min in a 2 M orthophosphoric acid solution. A beaker was filled quantitatively with the contents following a rinse with 25 mL of distilled water. After thorough mixing, the contents were filtered through ash-free filter paper (Whatman filter paper No. 1, provided by GE Healthcare, Tokyo, Japan). Test tubes were filled with 3 mL of TCA extract (filtrate) and 3 mL of TBA reagent (0.005 M), and the mixture was allowed to sit in the dark for 16 h. A blank sample was created by mixing 3 mL of 10% TCA and 3 mL of 0.005 M TBA reagent. The absorbance (O.D.) was measured at a fixed wavelength of 532 nm using a UV–VIS spectrophotometer (HITACHI, UV Spectrophotometer U-1800). The amount of malondialdehyde (in mg/kg) in the sample was the TBARS value, which was calculated by multiplying the O.D. value by the k factor of 5.2.

### 2.6 Immuno-antioxidant status

The serum enzymes like malondialdehyde (MDA) and superoxide dismutase (SOD) were measured using the techniques of Bradford (1976) and Kakkar et al. (1984), respectively. A commercial cortisol ELISA kit (Calbiotech Inc., U.S.A, CO368S) was used to evaluate the serum cortisol level. Using ELISA kits, the serum immunoglobulin {IgY (CK-bio-18160) and IgM (CK-bio-18163)} levels were ascertained in accordance with the manufacturer’s (Shanghai Coon Koon Biotech, China) technique. The weights of the slaughtered birds’ immune organs viz. bursa, thymus, and spleen, were recorded, and the splenic index was calculated.

### 2.7 Cecal microbial count

In order to generate a 10–1 dilution, 1 g of each cecal sample was homogenized in 9 mL of sterile saline peptone solution and agitated for 30 min. In pre-sterilized tubes, serial 10-fold dilutions were prepared up to 10^−6^. The media from Hi-media Laboratories Pvt. Ltd., Mumbai, were used. For coliforms, an aliquot of 0.1 mL of each dilution was distributed over MacConkey agar (370C for 24 h), and DeMan–Rogosa–Sharpe (MRS) agar (370C for 48 h) was used for lactic acid bacteria. The cecal contents were counted for coliform and *Lactobacillus* using the method outlined by Speck (1984), and values were expressed as log_10_ colony-forming units per gram (cfu/g).

### 2.8 Histomorphometric study

After being dehydrated and embedded in paraffin wax, the samples of jejunum fixed in 10% formalin were examined. Next, 6-µm-thick slices () were removed from the paraffin-embedded tissue blocks using a microtome and stained with hematoxylin and eosin. The measurement of crypt depth (CD) and villus height (VH) involved taking the mean of ten randomly selected portions from each sample using a computer-assisted morphometric system-equipped Nikon Eclipse Ni DS-Riz microscope. After that, the VH-to-CD ratio was computed appropriately (Thompson and Applegate, 2006).

### 2.9 Statistical analysis

One-way ANOVA was used to analyze the data. Duncan’s multiple-range test was used to compare the means. Each replication served as the experimental unit for comparing growth performance, while birds chosen from each replicate served as the experimental units for the other parameters. Significance was declared to be *P* < 0.05.

## 3 Results

### 3.1 Proximate composition of MSLP

The proximate analysis of *Malva sylvestris* leaf powder revealed the presence of 87.32% DM, 16.67% CP, 4.83% EE, 18.52% CF, and 6.34% TA.

### 3.2 Effect on growth performance

The results of growth performance in broilers supplemented with MSLP are presented in Table 2. Changes in the ADGs of broiler chickens did not reach significant levels by the inclusion of dietary treatments during the starter (1–3 weeks) and finisher (4–6 weeks) periods; however, a significant (*P* > 0.05) effect of treatments on ADG was observed during the overall period (1–6 weeks) compared to the control group. The lowest ADG was noticed in the control group (45.16 g/bird/day) and tended to improve in all other treatments. The highest improvement of 3.83% in the ADG was recorded in the T3 group fed 1.5% *Malva* powder in the diet compared to the control (*P* = 0.009). ADFI revealed significant (*P* < 0.05) differences during starter (1–3 weeks) and overall (1–6 weeks) periods between dietary treatments and control. The lowest ADFI during starter (52.91 g/bird/day) and finisher (78.51 g/bird/day) phases were noted in the T4 group compared with control and other dietary treatments. During the overall period, the F/G ratio reduced significantly (*P* = 0.048) in the T3 and T4 groups compared to control. The F/G ratio was significantly reduced (*P* = 0.048) in the T3 and T4 groups compared to control. The lowest feed/gain ratio was observed in the T3 group fed 1.5% MSLP in the diet.

**TABLE 2:** Effect of *Malva* supplementation on the performance of broiler chicken.

Parameter	T1	T2	T3	T4	SEM	*P-value*
ADG 1–3 weeks (g)	36.82	36.40	38.94	36.88	0.398	0.077
ADG 4–6 weeks (g)	67.77	68.32	69.89	68.50	0.318	0.080
ADG 1–6 weeks (g)	46.16^b^	46.29^b^	47.93^a^	46.55^b^	0.242	0.006
ADFI 1–3 weeks (g)	53.80^a^ ^b^	54.97^a^ ^b^	56.05^a^	52.91^b^	0.455	0.045
ADFI 4–6 weeks (g)	122.19	122.86	122.54	121.75	0.456	0.884
ADFI 1–6 weeks (g)	79.03^b^	79.75^a^ ^b^	80.46^a^	78.51^b^	0.271	0.025
F/G 1–3 weeks	1.46	1.51	1.44	1.44	0.018	0.475
F/G 4–6 weeks	1.80	1.80	1.75	1.78	0.012	0.387
F/G 1–6 weeks	1.71^b^	1.72^b^	1.68^a^	1.69^a^	0.008	0.048

T1 (Control)—Basal diet only; T2—Basal diet +1% MSLP; T3—Basal diet +1.5% MSLP; T4—Basal diet +2% MSLP; SEM—standard error of the mean.

^ab^ Means within a row bearing different superscripts differ significantly (*P* < 0.05). ADG—average weight gain; ADFI—average daily feed intake, F/G—feed/gain ratio.

### 3.3 Carcass and meat quality parameters

Tables 3, 4 reveal the effects of various dietary treatments on carcass and meat quality parameters in broiler chickens. A significant (*P* < 0.05) improvement was observed in slaughter and dressed weights in broilers fed 1.5% and 2% MSLP in the diet. Similarly, the breast yield was significantly (*P* < 0.05) higher in the T3 group, with no significant change (*P* > 0.05) in the thigh yield between various treatments and control. A significant (*P* < 0.05), dose-dependent decrease in the abdominal fat was observed with supplementation of MSLP up to 2% level in the diet compared to the control.

**TABLE 3:** Effect of supplementation of *Malva* on the carcass traits of broiler chicken.

Parameter	T1	T2	T3	T4	SEM	*P-value*
Slaughter weight (g)	2052.30^b^	2057.85^b^	2,113.32^a^	2094.35^a^	8.847	0.009
Dressed yield (%)	70.83^b^	70.92^b^	72.87^a^	72.74^a^	0.368	0.038
Breast weight (%)	30.66^b^	30.97^b^	32.87^a^	32.47^a^ ^b^	0.368	0.047
Thigh weight (%)	27.33	27.55	28.03	28.14	0.285	0.771
Abdominal fat (%)	1.21^a^	1.20^a^	1.18^a^ ^b^	1.13^b^	0.011	0.020

T1 (Control)—Basal diet only; T2—Basal diet +1% MSLP; T3—Basal diet +1.5% MSLP; T4—Basal diet +2% MSLP; SEM—standard error of the mean.

^ab^ Means within a row bearing different superscripts differ significantly (*P* < 0.05).

**TABLE 4:** Effect of *Malva* supplementation on the meat quality of broiler chicken.

Parameter	T1	T2	T3	T4	SEM	*P-value*
pH	6.09	5.97	5.93	5.89	0.041	0.402
WHC (%)	54.62	54.91	55.07	55.84	0.382	0.762
TBARS (mgMDA/kg)	0.53^a^	0.50^a^ ^b^	0.46^bc^	0.43^c^	0.014	0.026
L*	59.10	59.63	58.47	59.25	0.613	0.947
a*	5.95	5.97	5.86	5.89	0.114	0.991
b*	11.84	11.57	11.62	11.41	0.197	0.924

T1 (Control)—Basal diet only; T2—Basal diet +1% MSLP; T3-Basal diet +1.5% MSLP; T4-Basal diet +2% MSLP; SEM—standard error of the mean.

^abc^ Means within a row bearing different superscripts differ significantly (*P* < 0.05). WHC—water-holding capacity; TBARS—thiobarbituric acid-reactive substances. L*—lightness; a*—redness; b*—yellowness.

The differences in the pH and water-holding capacity (WHC) of breast meat were non-significant (*P* > 0.05) between control and various dietary treatments; however, thiobarbituric acid-reactive substances (TBARS) decreased significantly (*P* < 0.05) in the T3 and T4 groups. There was no significant difference (*P* > 0.05) in breast meat color between the dietary treatment groups and the control group.

### 3.4 Effect on serum antioxidants and cortisol

Table 5 depicts the impact of dietary MSLP supplementation on serum antioxidant levels and cortisol of broiler birds. Compared to the control, no significant difference (*P* > 0.05) was observed in SOD values among various groups; however, the levels of MDA decreased significantly (*P* < 0.05) in birds of the T4 group compared to other birds. The serum cortisol depicted significant (*P* < 0.05) enhancement in the T4 group compared to other groups.

**TABLE 5:** Effect of *Malva* supplementation on the serum antioxidants and cortisol of broiler chicken.

Parameter	T1	T2	T3	T4	SEM	*P-value*
SOD (U/mL)	147.23	154.62	149.47	158.05	3.140	0.673
MDA (nmol/mL)	7.96^a^	7.68^a^ ^b^	7.32^bc^	7.19^c^	0.105	0.008
Cortisol (U/mL)	15.69^b^	15.43^b^	16.09^b^	18.23^a^	0.405	0.023

T1 (Control)—Basal diet only; T2—Basal diet +1% MSLP; T3—Basal diet +1.5% MSLP; T4—Basal diet +2% MSLP; SEM—standard error of the mean.

^abc^ Means within a row bearing different superscripts differ significantly (*P* < 0.05). SOD—superoxide dismutase; MDA—malondialdehyde.

### 3.5 Effect on immune parameters

The effects of supplementing broiler chicken with MSLP on their immunological parameters are presented in Table 6. There was no significant (*P* > 0.05) effect on serum IgY levels between groups. Serum IgM values increased significantly (*P* < 0.05) in the T3 and T4 groups compared to other groups. The weight of immune organs, that is, bursa, thymus, and spleen, did not reveal any difference between control and various other treatments. Furthermore, no significant (*P* > 0.05) effect on the splenic index between control and dietary treatments was observed.

**TABLE 6:** Effect of *Malva* supplementation on the immune parameters of broiler chicken.

Parameter	T1	T2	T3	T4	SEM	*P-value*
IgY (µg/mL)	277.42	275.49	284.16	296.23	4.246	0.323
IgM (µg/mL)	511.46^b^	517.38^b^	531.25^a^ ^b^	539.14^a^	4.021	0.037
Bursa weight (g)	3.44	3.25	3.68	3.61	0.182	0.873
Thymus weight (g)	7.27	7.14	7.90	7.62	0.301	0.841
Spleen weight (g)	2.14	2.23	2.06	2.20	0.131	0.967
Splenic index	0.11	0.12	0.10	0.11	0.007	0.939

T1 (Control)—Basal diet only; T2—Basal diet +1% MSLP; T3—Basal diet +1.5% MSLP; T4—Basal diet +2% MSLP; SEM—standard error of the mean.

^ab^ Means within a row bearing different superscripts differ significantly (*P* < 0.05). IgY—immunoglobulin G; IgM—immunoglobulin M.

### 3.6 Effect on cecal microbiology

Table 7 illustrates the effects of MSLP supplementation on the cecal bacteriology of broiler chicks. Including *Malva* powder at a 2% level in the diet significantly (*p* < 0.05) decreased the coliform count compared to other groups. Furthermore, the *Lactobacillus* count increased numerically with the inclusion of dietary MSLP.

**TABLE 7:** Effect of *Malva* supplementation on the intestinal bacteriology of broiler chicken.

Parameter (cfu/g)	T1	T2	T3	T4	SEM	*P-value*
*Coliform*	6.53^a^	6.51^a^	6.48^a^ ^b^	6.37^b^	0.023	0.042
*Lactobacilli*	4.69	4.72	4.76	4.83	0.024	0.162

T1 (Control)—Basal diet only; T2—Basal diet +1% MSLP; T3—Basal diet +1.5% MSLP; T4—Basal diet +2% MSLP; SEM—standard error of the mean.

^ab^ Means within a row bearing different superscripts differ significantly (*P* < 0.05).

### 3.7 Effect on intestinal microstructure

**FIGURE 1:**
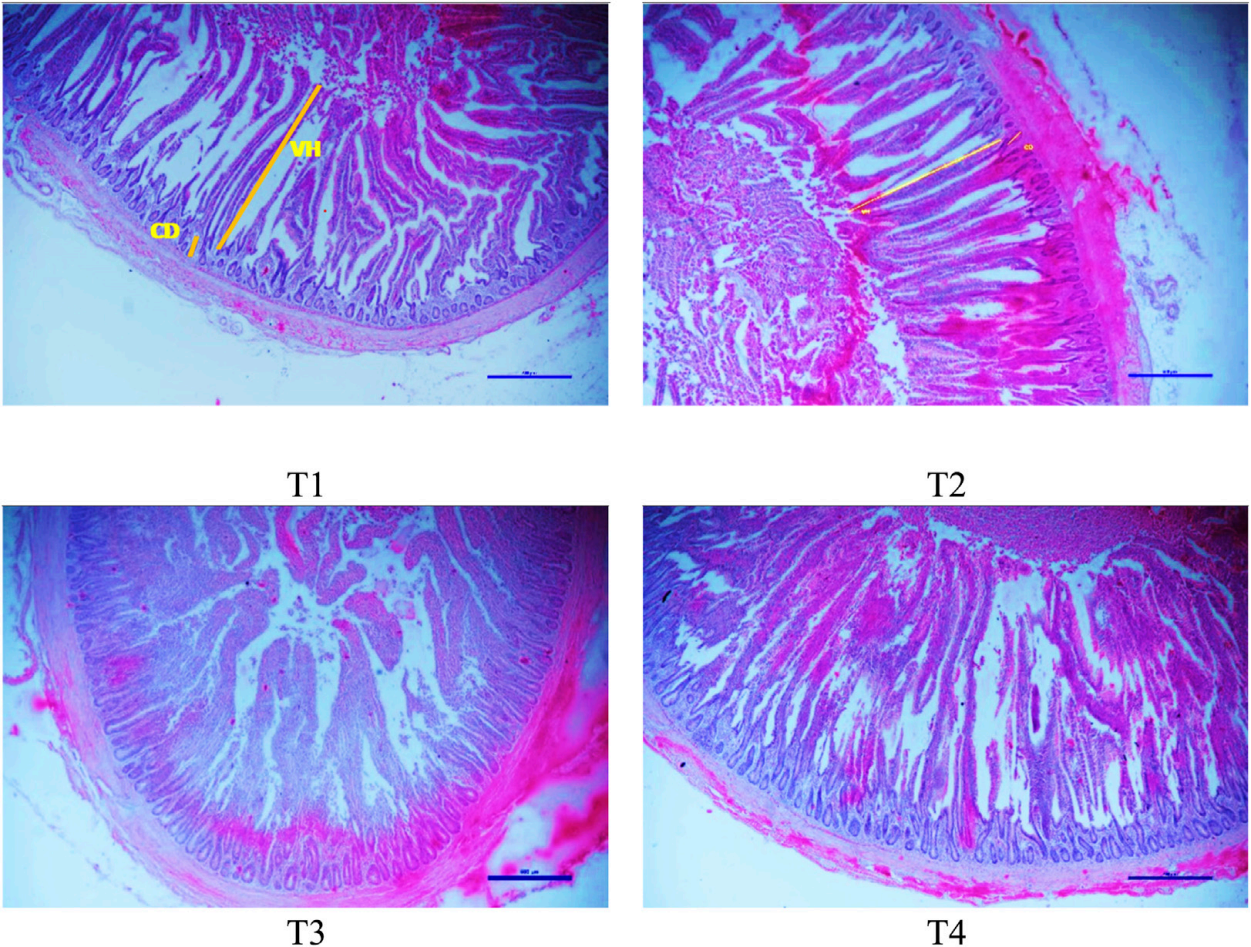
Representative histological micrographs of the jejunum in broiler chicken on day 42. The scale bar indicates 500 μm. T1 (Control)—Basal diet only; T2—Basal diet +1% MSLP; T3—Basal diet +1.5% MSLP; T4—Basal diet +2% MSLP. VH—villus height; CD—crypt depth.

Table 8; Figure 1 reveals the impact of dietary supplementation of MSLP on the jejunal histomorphology of broilers. The data showed that the diets supplemented with MSLP had no significant (*P* < 0.05) effect on VH and CD in the jejunum compared to the control group. However, the VH:CD ratio of broiler birds revealed significantly (*P* < 0.05) higher values in the T3 and T4 groups than in the control.

**TABLE 8:** Effect of *Malva* supplementation on the jejunal histomorphology of broiler chicken.

Treatment	1	2	3	4	SEM	*P-value*
VH (µm)	1,103.13	1,117.25	1,128.66	1,130.84	4.736	0.135
CD (µm)	171.27	169.53	159.98	157.49	2.022	0.014
VH:CD ratio	6.45^b^	6.59^b^	7.06^a^	7.19^a^	0.097	0.002

T1 (Control)—Basal diet only; T2—Basal diet +1% MSLP; T3—Basal diet +1.5% MSLP; T4—Basal diet +2% MSLP; SEM—standard error of the mean.

^ab^ Means within a row bearing different superscripts differ significantly (*P* < 0.05). VH—villus height; CD—crypt depth.

## 4 Discussion

The proximate analysis of *Malva sylvestris* powder revealed the presence of 87.32% DM, 16.67% CP, 4.83% EE, 18.52% CF and 6.34% TA. However, contrary to our results, Ahmed and Ibrahim (2016) reported higher CP (30.50%) and EE (21.9%), and lower CF (7.8%) in MSLP. This disparity in results could be attributed to the use of different Malva species, as well as environmental conditions such as soil fertility, moisture content, and growth temperature, all of which have a substantial impact on the nutritional value of plants grown in different geographical regions.

In our study, supplementation of Malva powder in the diet of birds improved ADG and F/G ratio especially at 1.5% level during the overall period (1-6 weeks). The results of our study are in line with others (Rahimian et al., 2021) who reported a significant improvement in weight gain and feed efficiency following supplementation with MSLP up to 70 mg/kg diet in broiler chicken. Kiani et al. (2015) reported a non-significant increase in the body weight and feed efficiency of broilers on addition of Malva at 200 mg/kg diet. Similarly, Kadam Abed Ameer and Mehmood (39) documented an improvement in overall weight gain and feed conversion ratio in broiler birds using *Malva parviflora* leaves as the herbal additive in their diet. However, in contrast to our results, Soltani and Nobakht (2016) reported that supplementation of Malva at 2% level in the diet produced no significant effect in improving the weight and feed efficiency of broiler birds. The positive effect in the ADG and F/G ratio could be attributed to the synergistic impact of various bioactive compounds in phytobiotics (Hussein et al., 2020). Phytobiotics have been known to promote intestinal architecture, increase villus length, and improve nutritional absorption (Tabatabaei, 2016). Moreover, phytobiotics increase the secretion of digestive enzymes, decrease gut infections, and increase lactobacillus spp. populations (Adil et al., 2024; Alloui et al., 2014). All of these factors contribute to better production performance of birds.

The ADFI of broiler birds in our study revealed that feed intake decreased by increasing the level of MSLP in the diet of broilers. A significant reduction in FI on addition of *Malva parviflora* leaf powder in the diet of Japanese quail has been reported by Ahmed and Ibrahim (2016). In contrast, Kiani et al. (2015) reported an increase in feed intake during 3rd and 6th week of experiment on addition of Malva up to 400 mg/kg diet of broilers. Rahimian et al. (2021) also documented an increase in feed intake by supplementation of Malva powder up to 70 mg/kg diet in broilers. The decrease in FI at higher levels of MSLP could be attributed to the fact that phytogenics at higher dose reduce palatability due to the bitter taste (Sugiharto, 2021).

In the present study, better slaughter weight, dressed weight and breast yield was observed on supplementation of MSLP at 1.5% level. These results were partly in consistent with Rahimian et al. (2021), who reported a significant improvement in pre-slaughter weight on supplementation of Malva powder up to 70 mg/kg broiler diet with no significance in dressed yield. A non-significant increase in pre-slaughter weight, dressed weight and breast yield of broilers on addition of Malva at 400 mg/kg of diet has been documented (Kiani et al., 2015). In our study, a significant decrease in the abdominal fat (%) was observed on increasing the level of MSLP supplementation up to 2% level in the diet, which corroborates with the findings of previous researchers (Rahimian et al. (2021) who also reported the similar results.

Phytogenic feed additives are known to improve the meat quality which could be due to antimicrobial and antioxidant activities that may lead to reduced lipid oxidation and microbial load of carcass (Aksit et al., 2006; Stanacev et al., 2011). Moreover, Soltan et al. (2008) reported that adding essential oils, herbs, or spices to poultry products decreases bacteria in the products, ensuring safe food and quality preservation of raw and cooked meat. In the present study, pH and WHC values revealed no significance on dietary supplementation of Malva powder, however a significant reduction was observed in TBARS as the level of Malva powder was increased especially at 2% level in the diet. The TBARS of meat shows the degree of lipid peroxidation. Since, poultry meat contains a high concentration of unsaturated fatty acids, it oxidizes at a faster rate than other varieties of meat. The TBARS method is used to quantify the scale of rancidity and souring caused by oxidation in fat and fatty sections of meat, and it is based on the production of compounds from oxidative damage such as MDA (Tekce et al., 2020). In our study, the reduction in TBARS depicts beneficial effects of MSLP supplementation in the diet of broilers. According to Kuttappan et al. (2012), consumers' preferences for meat are largely influenced by its colour. Meat colour is associated with its freshness, attractiveness and consumer acceptability. The colour coordinates of meat were found well within the normal range indicating no adverse effect of MSLP supplementation in the broiler diet.

There was no significant rise in SOD levels, however and a significant drop in MDA following dietary inclusion of MSLP was found, particularly at 2% dose. This revealed the antioxidant effect of MSLP supplementation in broilers. SOD protects intracellular lipids from peroxidation, whereas MDA measures lipid peroxidation (Moustafa et al., 2020). Wei and Shibamoto (2007) investigated the antioxidant properties of phytogenic substances. The majority of antioxidant activity seen in phytogenic substances have been attributed to the phenolic and terpene components present in them.

According to Uyanga et al. (2023), stress is any state that adversely impacts an animal's basic functions. Animals can experience physiological stress due to a variety of conditions, including extreme temperatures, fast growth rates, overcrowding or excessive stocking density, starvation, and others. Chronic stress has been linked to decreased productivity and possibly death (Gebregeziabhear et al., 2015). Serum cortisol concentration is a routine and often measured biomarker in birds to determine the level of stress (Weimer et al., 2018; Freeman and Newman, 2018; Will et al., 2019). As the level of MSLP was increased, a significant drop in the blood cortisol concentration was observed in birds indicating the anti-stress effect of MSLP. Phytobiotics have been found to protect the poultry birds against environmental stresses and boost their immune system (Song et al., 2018).

Chicken serum has long been known to contain two classes of immunoglobulins, IgY and IgM (Leslie and Clem, 1969). In the current study, there was increase in the serum concentration of IgM in the birds fed MSLP in the diet, however the IgY values remain non-significant between treatments and control. These results could be attributed to the fact that phytogenics exert immunomodulatory effects by increasing immune cell proliferation, cytokine expression, and antibody titers (Lee et al., 2010; Park et al., 2011; Pourhossein et al., 2015). However, no effect was observed on the weight of bursa, thymus, spleen or splenic index among various treatments and control.

The gut microbiome includes both beneficial bacteria like Lactobacilli and Bifidobacteria and harmful bacteria like E. coli, Campylobacter, and Salmonella (Li et al., 2009). Phytogenic compounds are known to alter gut microbiota in a positive manner, thereby improving host health (Tollba et al., 2012). Several studies have demonstrated that phytogenic compounds or extracts can lower the population of pathogenic bacteria and their metabolites while increasing the growth of beneficial microflora, hence protecting birds from various ailments (Liu et al., 2014). In the present study, significant reduction in the cecal coliform count and a non-significant increase in lactobacilli count were recorded as the level of MSLP was increased in the diet of broilers. These results are in agreement with others (Rahimian et al., 2021) who reported a significant reduction in E.coli count and an increase in lactobacilli count in broiler birds on supplementation of malva powder in the diet. Additionally, Alagbe (2021) reported that phytochemical substances like alkaloids, flavonoids, and phenols can promote the growth of lactobacillus species and reduce the activities of pathogenic bacteria through competitive exclusion.

In the current study, supplementation of MSLP at 1.5 and 2% level in the diet revealed a numerical increase in VH and a significant enhancement in the VH:CD ratio in broilers. These results were partly in consistent with previous researchers (Kiani et al., 2015) who also reported an increase in VH and VH:CD ratio on supplementation of broilers with Malva up to 400 mg/kg of diet. Longer villi with more surface area can result in higher feed utilisation, growth performance, and bird health (Pluske et al., 1996), thus justifying the improvement in the ADG of birds in groups fed MSLP at 1.5 and 2% level. Previous studies have shown that supplementation of herbal feed additives can alter the histological structure of the intestine including an elevation of the intestinal villi by deepening of its crypts (Steiner, 2006; Murali et al., 2012).

## 5 Conclusion

The present study revealed an increase in the average daily gain and feed efficiency of broilers fed *Malva sylvestris* leaf powder (MSLP) in the diet, especially at the 1.5% level. The positive impact of *Malva* may be attributed to the beneficial effects on the carcass traits, immunological parameters, serum antioxidants, intestinal microbiology, and gut histomorphology of broiler birds. Based on the current findings, *Malva sylvestris* leaf powder at a concentration of 1.5% and 2% could be utilized as a feed supplement to improve the production performance of broiler chicken.

## Data Availability

The original contributions presented in the study are included in the article/Supplementary Material; further inquiries can be directed to the corresponding author.
